# Enhanced West Nile virus surveillance in the North Kent marshes, UK

**DOI:** 10.1186/s13071-015-0705-9

**Published:** 2015-02-10

**Authors:** Alexander GC Vaux, Gabriella Gibson, Luis M Hernandez-Triana, Robert A Cheke, Fiona McCracken, Claire L Jeffries, Daniel L Horton, Simon Springate, Nicholas Johnson, Anthony R Fooks, Steve Leach, Jolyon M Medlock

**Affiliations:** Medical Entomology group, MRA, Emergency Response Department, Public Health England, Porton Down, Salisbury, SP4 0JG UK; Natural Resources Institute, University of Greenwich at Medway Central Avenue, Chatham Maritime, Kent, ME4 4TB UK; Animal and Plant Health Agency, Wildlife Zoonoses and Vector-Borne Diseases Research Group, Woodham Lane, Addlestone, Surrey KT15 3NB UK; Current address: Department of Disease Control, London School of Hygiene and Tropical Medicine, Keppel Street, London, WC1E 7HT UK; School of Veterinary Medicine, University of Surrey, Guildford, UK; Institute of Infection and Global Health, University of Liverpool, Liverpool, L69 7BE UK; Microbial Risk Assessment and Behavioural Science, Emergency Response Department, Public Health England, Porton Down, Salisbury, SP4 0JG UK; NIHR Health Protections Research Unit in Emerging and Zoonotic Infections, Porton Down, UK

**Keywords:** *Culex modestus*, United Kingdom, West Nile virus, Usutu virus, Culicidae, Mosquito, Surveillance

## Abstract

**Background:**

As part of efforts to more fully understand the potential risks posed by West Nile virus (WNV) and Usutu virus (USUV) in the UK, and following on from previous reports of a potential bridge vector *Culex modestus* for these viruses, at wetland sites in North Kent, mosquito surveillance was undertaken more widely across the Isle of Sheppey, the Hoo Peninsula and the Kent mainland.

**Methods:**

Larval surveys were conducted and Mosquito Magnet® adult traps were used to collect adult mosquitoes. Pools of female mosquitoes were tested for the presence of WNV using real-time reverse transcriptase polymerase chain reaction. A subset of samples was tested for USUV.

**Results:**

*Culex modestus* was found in both the pre-imaginal and imago stage at all five locations surveyed, accounting for 90% of adult mosquitoes collected. WNV or USUV were not detected in any sample.

**Conclusions:**

Although no mosquitoes have been shown to be virus positive, the field survey data from this study demonstrated the dominance of an important bridge vector species for WNV in this region. Its wide geographical distribution highlights the need to update risk assessments on WNV introduction, and to maintain vigilance for WNV in the South East of England.

## Background

West Nile virus (WNV) has previously been identified as a vector-borne pathogen of concern to UK public and veterinary health [1,2]. The presence of WNV in the UK has not been recorded, despite surveillance in humans, horses, and wild birds [3,4], but serological studies of resident and migratory birds have reported virus-specific neutralizing antibodies to WNV, Usutu virus (USUV) and Sindbis viruses (SINV) [5]. Transmission of the virus to humans and horses is reliant upon competent bridge vectors transmitting the virus from an enzootic bird-mosquito-bird cycle to bird-mosquito-human/horse transmission, where humans and horses are dead-end hosts [6,7]. Thirty four species of mosquito have been recorded in the British Isles, nine of which have been implicated in WNV transmission elsewhere [1,2,8]. The main competent bridge vectors in continental Europe are *Culex pipiens molestus, Cx. perexiguus* and *Cx. modestus* [9,10]. Of these taxa, the UK has localised populations of *Cx. pipiens* biotype *molestus*, which can be a human biting nuisance and *Cx. modestus.* However, there are no populations of *Cx. perexiguus* which currently has a distribution restricted to warmer climates in the Mediterranean, North Africa and Asia. Until recently, *Cx. modestus* had not been recorded in the UK since the 1940s when three adults and ten larvae were found and eradicated in Portsmouth [11]; however, it has recently been reported in significant numbers at three locations in North Kent [12], and also recorded in lower numbers Cambridgeshire and Dorset [13].

Golding *et al.* [12] identified the presence of *Cx. modestus* at three sites on the Hoo Peninsula in North Kent, and the species was found in significant numbers both as larvae and as trap-caught adults. A total of 679 *Cx. modestus* adults were collected over twenty trap nights at Northward Hill, on the Hoo Peninsula, (April-October), at a mean count per night of 33.95, representing 75% of the total catch. *Culex modestus* is considered to be the principal vector of WNV in parts of Europe, where it is found in a range of wetland habitats including reedbeds and rice fields, and is known to aggressively feed on birds, and mammals including humans [14,15] The occurrence of this species in the North Kent marshes in habitats frequented by migratory birds and grazing horses is a consideration when conducting surveillance for WNV. Furthermore, a principal enzootic vector, *Cx. pipiens pipiens* is common in the UK, and therefore the co-existence of these two species in North Kent would increase the risk for transmission of the virus should it occur there, to horses and humans if WNV were introduced.

The study aimed to confirm the persistence and map the extent of the distribution of *Cx. modestus*, and combine ongoing entomological surveillance [12,13] and WNV surveillance in wildlife [3] to better inform the risk assessment and identification of risk areas.

## Methods

### Mosquito survey

Following previous surveys that identified the presence of the vector *Cx. modestus* at Elmley Marshes (51°22′25”N, 0°46′51”E), Northward Hill Nature Reserve (51°23′47”N, 0°42′36”E), and Cliffe Marshes (51°27′48”N, 0°33′2”E) [12], a site visit of potential larval habitats was conducted during 2012 using maps and field visits. In May 2013 an initial field survey was conducted to identify sites across North Kent, and nine sites were chosen for larval surveys: the previously surveyed sites at Cliffe Marshes, Northward Hill and Elmley Marshes, and additional sites at Allhallows Marshes (51°27′60”N, 0°39′19”E), Chetney Marshes (51°27′48′N, 0°33′2”E), Oare Marshes (51°20′34”N, 0°53′20”E), Graveney Marshes (51°20′5”N, 0°55′55”E), and the Harty Marshes (51°22′1”N, 0°55′12”E). After a further larval survey at Allhallows Marshes and Harty Marshes it was decided not to take samples at these locations due to the absence of suitable aquatic habitats. Furthermore, owing to difficult access at Graveney Marshes, no further surveys were conducted there.

**Figure 1:**
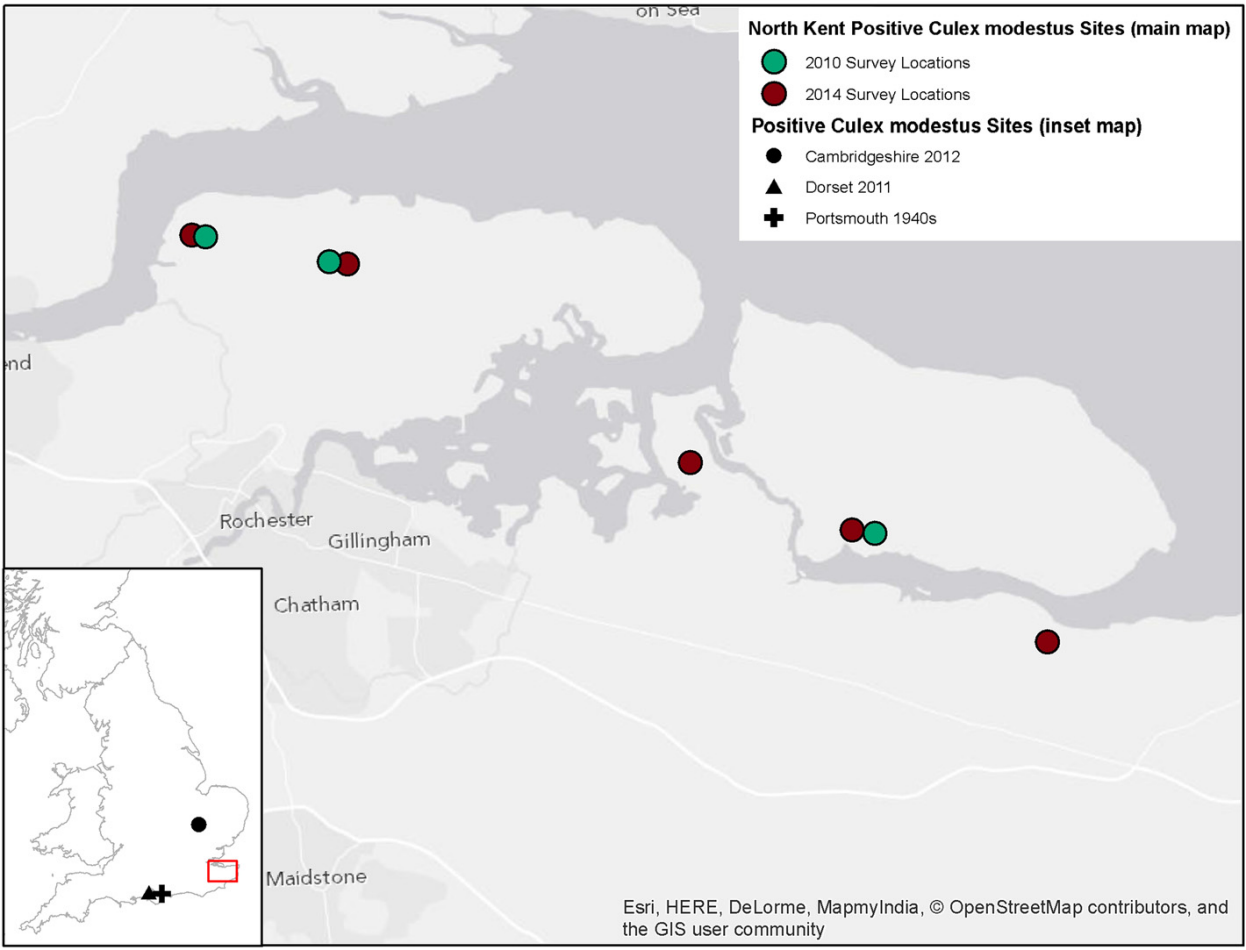
**Map of the survey locations and historical records.**

Larval surveys were conducted at the remaining five sites (Figure 1) every two weeks from 1^st^ July 2013 to 19^th^ August 2013. Approximately 25 larval sampling points were chosen at each site. Three 250 ml dips were taken at each sampling point and pre-imaginal stages (I-III, IV instar larvae, pupae) were collected and identified using the keys of Schaffner *et al.* [16]. No attempt was made to differentiate between *Cx. pipiens* s.l. and *Cx. torrentium,* as larvae were not reared to IV instar, and males were not collected. Therefore, *Cx. pipiens* s.l. and *Cx. torrentium* are referred to as *Cx. pipiens* s.l./*Cx. torrentium.* The *Anopheles maculipennis* species complex was not identified further to species (referred to as *An. maculipennis* s.l.) which would have required DNA analyses. The latter was not deemed necessary given that this project was focused on *Cx. modestus.*

Adult trapping was conducted using the Mosquito Magnet® Executive Mosquito trap (MosquitoMagnet, Lititz, Pennsylvania, USA; http://www.mosquitomagnet.com/) with Octenol (MosquitoMagnet, Lititz, Pennsylvania, USA; http://www.mosquitomagnet.com/). This trap was chosen given its proven ability to collect large numbers of the target species, *Cx. modestus* [12] and the ability of the trap to run for a number of nights without interruption, servicing or maintenance. It has also been proven to collect large numbers of British mosquitoes [17,18]. Other traps including CO_2_ light traps were considered, but given their reliance on batteries and the frequency required to service them, they were not chosen. Traps were run from Monday to Thursday (3 nights) every other week at Cliffe Marshes, Northward Hill, Chetney Marshes, Elmley Marshes and Oare Marshes. Traps at Cliffe and Northward Hill ran on alternate weeks from week 29–37, and traps at Chetney, Elmley and Oare Marshes ran on alternate weeks from week 30–38. Two traps were run at Elmley given the size of the site and the confirmed presence of the species in previous surveys. Adult mosquitoes were removed from traps, placed on dry ice and transported to the laboratory to be identified on a cold plate of dry ice. Samples were kept at −80°C until viral testing. Mosquito abundance was calculated per litre for larvae (L^s^), and per trap night for adults, and expressed as mean number of adults per night over the season (n^s^).

### Virus testing

Adult females of target species were separated in pools of ten specimens per tube whenever possible, placed in disruption tubes and sent to the Animal and Plant Health Agency (APHA) for molecular analysis for virus detection. Tissue disruption of the whole specimen, homogenization and RNA extraction was undertaken using a Qiagen RNeasy Mini Kit. Two pre-treated 5 mm stainless steel beads were placed in each 2 ml disruption microtube containing the pooled sample. Each tube was homogenized dry for 3 min at 25Hz in the TissueLyser (Qiagen) and then centrifuged for 3 min at full speed (12,100 x g). Half of the pellets that formed were placed in cell culture medium (Medium (E-MEM/10%FBS) for cell culture/virus isolation, if needed. Immediately 600μl of buffer RLT (Qiagen) was dispensed to each tube, vortexed and centrifuged. Without disturbing any visible pellet, the supernatant was transferred from the disruption tube to a 1.5 ml collection tube and kept at −20°C. RNA was extracted from mosquito homogenates using Qiagen RNeasy Mini kit as per the manufacturer’s instructions. RNA was eluted in 50 μl of nuclease-free water.

Mosquito RNA samples were screened for the presence of WNV and USUV virus using 2 μl of the total RNA extract and employing probe-based PCR techniques in Mx3000P real time PCR systems (Stratagene). Published methods for the detection of WNV RNA were followed [19]. This primer set amplifies a conserved region of the 5′-UTR and part of the capsid gene producing a product of approximately 144 base pairs. The RT-PCR was carried out in a 50 μl reaction volume containing: nuclease-free water; 2× RT-PCR reaction mix for probes (BioRad); WNV Linke FOR primer (5pmol) (1 μl); WNV Linke REV primer (5pmol) (1 μl); WNV Linke probe (2.5pmol) (1 μl); and iScript RT for one step RT-PCR (BioRad). PCR thermal amplification conditions were used as previously published [19]. A no-template control and a WNV positive RNA sample (strain goose Israel 1998) were included on every test plate.

For the detection of USUV RNA the primers and probe of Jöst *et al.* [20] were used. These oligonucleotides are directed at the NS1gene of USUV and amplify a fragment approximately 91 base pair in length. The RT PCR was carried out in a 25 μl reaction volume containing: RNase-free water; 2x QuantiTect RT-PCR Master mix; Jost USUV Primer mix (10 μM primer/1.25 μM probe) and QuantiTect RT mix (Qiagen). A no-template control and a USUV positive RNA sample (strain Arb153) were included on every test plate.

## Results

### Mosquito survey

Eleven species (*Anopheles claviger, An. maculipennis* s.l., *An. plumbeus, Coquillettidia richiardii, Ochlerotatus caspius, Oc. dorsalis, Oc. detritus, Oc. flavescens, Culex pipiens* s.l./*Cx. torrentium*, *Cx. modestus* and *Culiseta annulata*) were identified in the six adult traps across the five sites. *Culex modestus* was found at all traps and at all larval sites.

In total, 5724 adults were trapped over 75 trap nights at a mean abundance of 19.07/n^s^ (Table 1). *Culex modestus* was the most abundant species (5216/5724; 91%; 17.39/n^s^), thereafter *Cq. richiardii* (n = 220, 0.73/n^s^), *Cx. pipiens* s.l./*Cx. torrentium* (n = 164, 0.54/n^s^) and *An. maculipennis* s.l. (n = 46, 0.15/n^s^). The remaining species were trapped in lower numbers: *An. claviger* (0.12/n^s^)*, Oc. flavescens* (0.05/n^s^), *Oc. detritus* (0.03/n^s^)*, Cs. annulata* (0.03/n^s^), *Oc. dorsalis* (0.01/n^s^)*, Oc. caspius* (0.003/n^s^)*, An. plumbeus* (0.003/n^s^).

**Table 1:** **Adult data shown for species (** ***Cx. modestus, An. maculipennis*** **s.l.** ***, Cx. pipiens*** **s.l./** ***Cx. torrentium, Cq. richardii*** **), and sites (Chetney, Cliffe, Elmley Field and Elmley Barn, Northward Hill, and Oare), shown by mean number of adults per night over the season (n** ^**s**^ **)**

**Site**	**Total number adult females**	**All species (n** ^**s**^ **) ± SE**	**Mean** ***Cx. modestus*** **(n** ^**s**^ **) ± SE**	**Mean** ***An. maculipennis*** **s.l. (n** ^**s**^ **) ± SE**	**Mean** ***Cx. pipiens*** **s.l./** ***Cx. torrentium*** **(n** ^**s**^ **) ± SE**	**Mean** ***Cq. richiardii*** **(n** ^**s**^ **) ± SE**
Chetney	918	16.11 ± 7.57	12.75 ± 7.70	0.02 ± 0.02	2.40 ± 1.70	0.68 ± 0.46
Cliffe	2795	44.4 ± 24.1	41.90 ± 24.3	0.05 ± 0.03	0.03 ± 0.03	2.00 ± 1.61
Elmley barn	922	22.0 ± 15.5	21.2 ± 15.6	0.31 ± 0.18	0.14 ± 0.14	0.19 ± 0.17
Elmley field	179	5.97 ± 4.28	4.97 ± 4.39	0.37 ± 0.21	0.33 ± 0.17	0.30 ± 0.30
Northward	686	13.34 ± 7.57	12.55 ± 7.65	0.28 ± 0.13	0.06 ± 0.04	0.49 ± 0.47
Oare	222	3.89 ± 1.39	2.98 ± 1.47	0.07 ± 0.04	0.11 ± 0.08	0.23 ± 0.16
**All sites**	**5724**	**19.07 ± 5.91**	**17.39 ± 5.95**	**0.15 ± 0.04**	**0.55 ± 0.38**	**0.73 ± 0.36**

**Figure 2:**
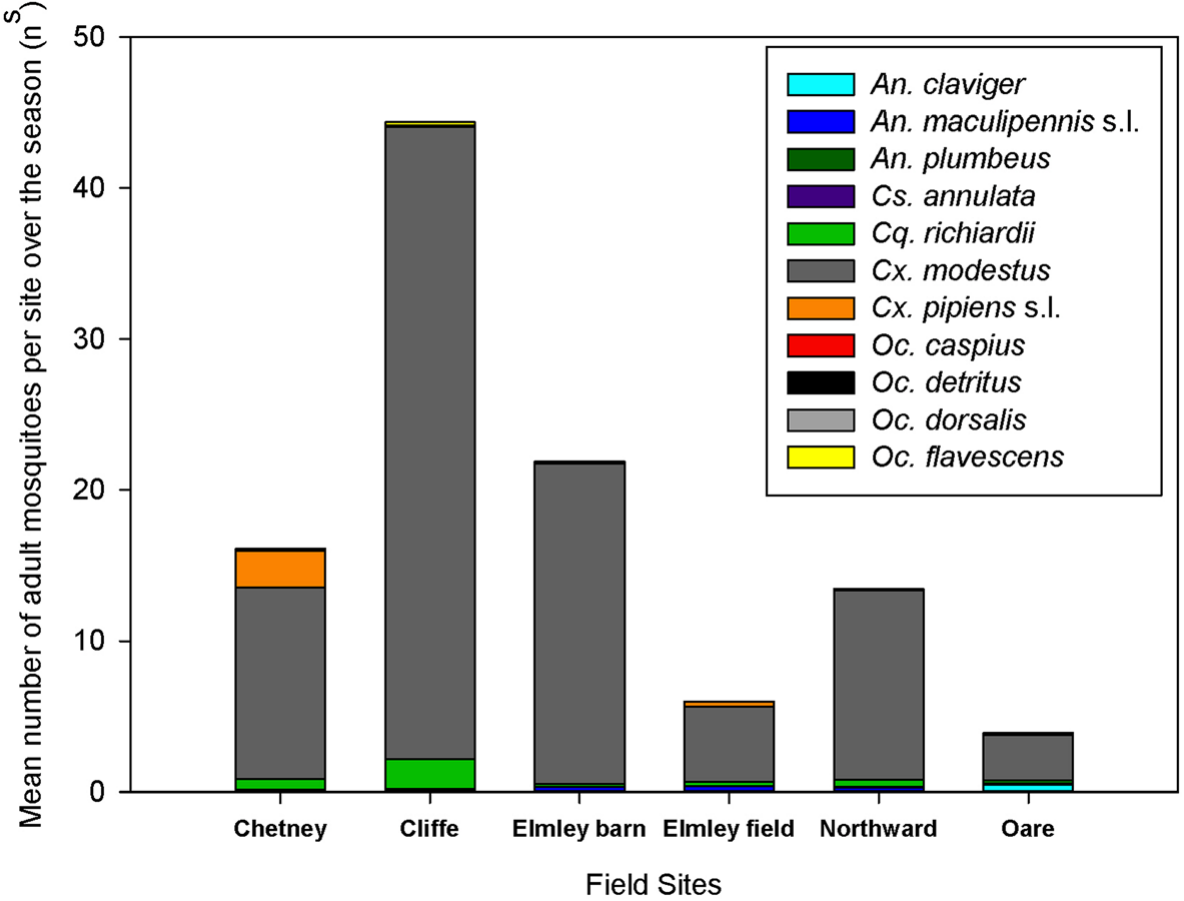
**The abundance of adult mosquitoes by species at Chetney, Cliffe, Elmley, Northward Hill and Oare Marshes.** Mosquito Magnets were run for three nights during five weeks from July to September 2013.

**Figure 3:**
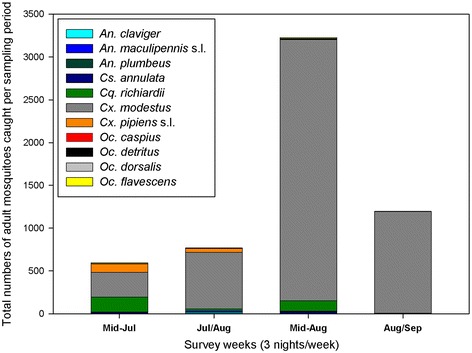
**The abundance of adult mosquitoes by species shown by temporal categories: Mid-Jul; Jul/Aug; Mid-Aug, and Aug/Sep.**

The highest adult abundances across the season were found at Cliffe Marshes (44.4/n^s^), Elmley Barn (22.0/n^s^), Chetney (16.11/n^s^) and Northward Hill (13.45/n^s^), with lower adult abundances at Elmley field (5.97/n^s^) and Oare (3.89/n^s^) (Figure 2).The proportion of mean adult *Cx. modestus* per trap night to total mean adults trapped per night ranged between 76% and 96% across the sites. The greatest abundances of adult mosquitoes were found during the mid-August trap weeks (Figure 3).

**Figure 4:**
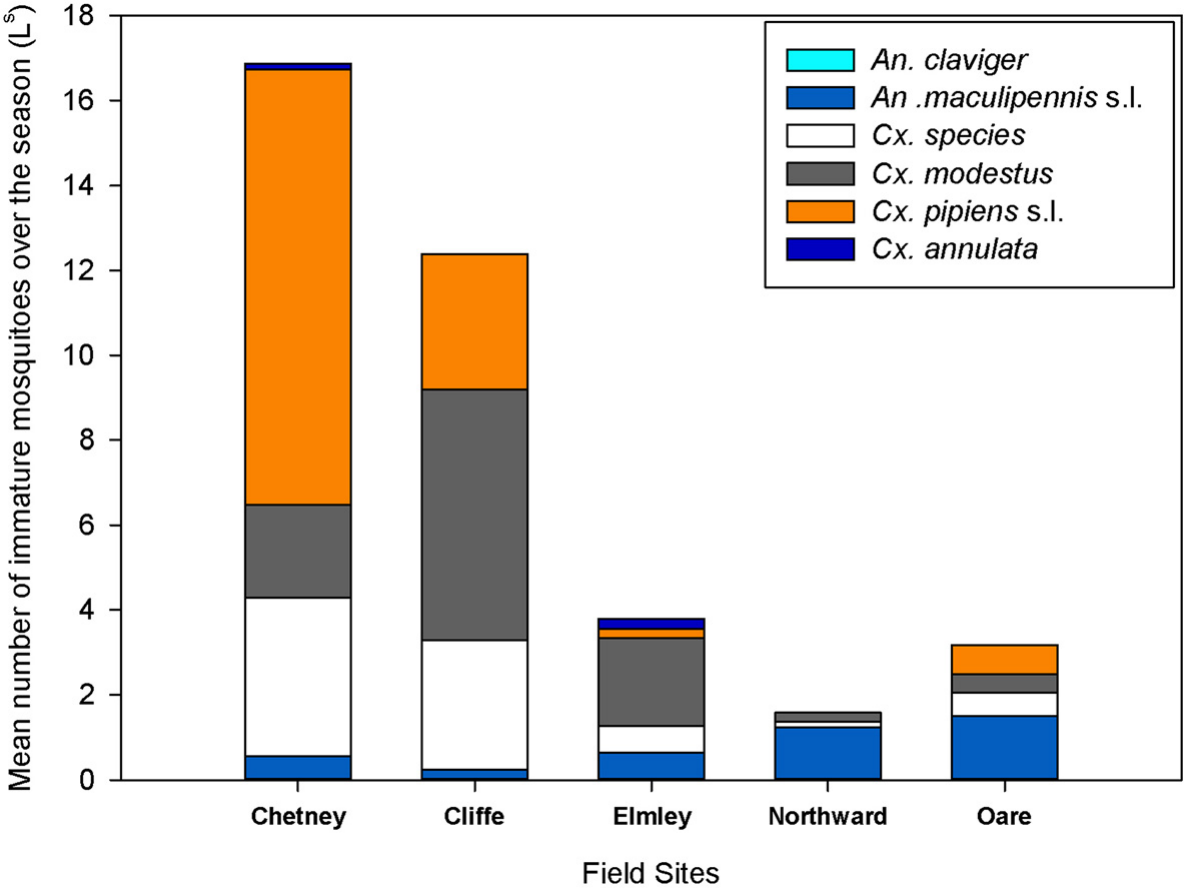
**The abundance of immature mosquitoes by species at Chetney, Cliffe, Elmley, Northward Hill and Oare Marshes.**

The larval surveys yielded five species: *An. claviger, An. maculipennis* s.l.*, Cx. modestus, Cx. pipiens* s.l*./Cx. torrentium,* and *Cs. annulata* (Figure 4)*.* Over the course of the survey, 7.36/L^s^ (3945 total larvae) were collected, dominated by two species *Cx. modestus* (7.36/L^s^) and *Cx. pipiens* s.l./*Cx. torrentium* (2.53/L^s^)*.* A third classification of *Culex* sp. (1.56/L^s^) was made on account of the difficulty in separating I & II instar larvae of this genus. *Anopheles maculipennis* s.l. (0.80/L^s^) was more abundant than *An. claviger* (0.004/L^s^), and *Cs. annulata* (0.086/L^s^).

The highest immature abundances across all species were reported at Chetney marshes (17.05/L^s^) and Cliffe marshes (12.48/L^s^), with lower abundances at Elmley marshes (3.81/L^s^), Oare marshes (3.18/L^s^) and Northward Hill (1.64/L^s^). The highest mean number of *Cx. modestus* was recorded at Cliffe and Chetney Marshes (Table 2).

**Table 2:** **Larvae data shown for species (** ***Cx. modestus, An. maculipennis*** **s.l.** ***, Cx. pipiens*** **s.l./** ***Cx. torrentium, Cx*** **. species), and sites (Chetney, Cliffe, Elmley, Northward Hill and Oare)**

**Site**	**Number sampling points (3×250ml)**	**Water surveyed (litres)**	**Total number larvae**	**Mean larvae per litre (L** ^**s**^ **) ± SE**	**Mean** ***Cx. modestus*** **per litre (L** ^**s**^ **) ± SE**	**Mean** ***An. maculipennis*** **s.l. per litre (L** ^**s**^ **) ± SE**	**Mean** ***Cx. pipiens*** **s.l./** ***Cx. torrentium*** **per litre (L** ^**s**^ **) ± SE**	**Mean** ***Cx.*** **species per litre (L** ^**s**^ **) ± SE**
Chetney	114	85.5	1458	17.05 ± 5.51	2.20 ± 0.62	0.55 ± 0.17	10.25 ± 4.33	3.73 ± 3.56
Cliffe	154	115.5	1442	12.48 ± 2.04	5.91 ± 1.74	0.24 ± 0.06	3.20 ± 0.88	3.04 ± 1.02
Elmley	193	144.75	552	3.81 ± 0.50	2.07 ± 0.46	0.63 ± 0.17	0.22 ± 0.06	0.62 ± 0.22
Northward	97	72.75	119	1.64 ± 0.31	0.21 ± 0.10	1.22 ± 0.30	0.00 ± 0.00	0.14 ± 0.09
Oare	157	117.75	374	3.18 ± 0.34	0.43 ± 0.12	1.50 ± 0.28	0.69 ± 0.24	0.55 ± 0.15
**All sites**	**715**	**536.25**	**3945**	**7.36 ± 1.01**	**2.31 ± 0.41**	**0.80 ± 0.09**	**2.53 ± 0.73**	**1.56 ± 0.61**

### Virus testing

The RT PCR for WNV did not detect the presence of WNV RNA in any *Culex modestus* samples (282 pools; 2290 female specimens; Table 3). In addition, a panel of 125 samples of *Cx. modestus* (1025 females) was also tested to detect the presence of USUV virus RNA. There was no product amplification of USUV RNA in any sample. The presence of WNV was also tested in a further eight species (24 pools; 113 specimens; Table 3). In all samples RNA was not detected by real time RT-PCR.

**Table 3:** **Number of genera, species and specimens of female** ***Culex modestus*** **and other mosquito species tested for WNV and USUV collected at Kent marshes**

**Species**	**Pools**	**No. of mosquitoes**
*An. claviger*	3	8
*An. maculipennis* s.l*.*	3	9
*Cq. richardii*	8	55
*Cs. annulata*	2	3
*Cx. modestus*	282	2290
*Cx. pipiens* s.l./*Cx. torrentium*	1	2
*Oc. caspius*	1	1
*Oc. dorsalis*	1	1
*Oc. flavescens*	5	34
**All species**	**306**	**2403**

## Discussion

*Culex modestus* was identified at all sites where larval and adult surveys were conducted; it was found to be the dominant species trapped by the Mosquito Magnet® at all of the sites, and in larval surveys it was proven to be as abundant as the ubiquitous species *Cx. pipiens* s.l./*Cx. torrentium. Culex modestus* dominated the species composition trapped at Northward Hill, representing 93% of the total adults caught, which is even higher than the results of adult trapping during 2010 when the species accounted for 75% of the total adult catch [12]. In a previous study, comparing the CDC light trap and Mosquito Magnet traps at Elmley Marshes, *Cx. modestus* was not recorded, however *Cx. pipiens* s.l. and *Cx. torrentium* were [17].

The Mosquito Magnet® uses Octenol lures, which selectively attract mammal-biting species, and explains the lack of *Culex pipiens* s.l./*Cx. torrentium* adults caught at traps where larval surveys indicated a high population of the species. The trap at Chetney was the only trap to catch significant numbers of *Cx. pipiens* s.l./*Cx. torrentium* (137 adults over the season; < 10 adults at all other sites), which is an unusual finding, and differs from previous results when no *Cx. pipiens* s.l./*Cx. torrentium* were trapped at the Isle of Sheppey using Mosquito Magnets [17]. Mosquito Magnets have been used extensively in habitats with high densities of *Cx. pipiens* s.l./*Cx. torrentium* across the UK and very low numbers of this species have been trapped when using the Octenol lure [18]. This may warrant further investigation of the species trapped here, as it could be a hybrid form of the ornithophagic *Cx. pipiens* biotype *pipiens* and the anthropophagic stenogamic *Cx. pipiens* biotype *molestus*. However it was noted that there was a partridge feeder nearby to the Mosquito Magnet, and therefore it is possible that the *Cx. pipiens* s.l./*Cx. torrentium* were inadvertently drawn into the trap. It is worth noting that the distribution of *Cx. torrentium* in the UK is poorly understood due to its close morphological similarity to *Cx. pipiens* s.l., and given that *Cx. torrentium* has also been implicated as an important WNV vector, further studies to more fully understand this species are needed in the UK.

Through the use of rapid, specific RT-PCR assays we were unable to detect WNV or USUV RNA within any of the mosquito samples tested. This corroborates the absence of WNV through surveillance in birds, conducted by APHA (formerly Animal Health and Veterinary Laboratories Agency) since 2001 [3,4]. However it should be noted that in regions with high circulation of WNV, many thousands of mosquitoes are routinely tested, and therefore the numbers of samples tested in this study are relatively low for this type of virus testing. Further virus surveillance will need to maximise the number of mosquitoes made available for testing and methods to streamline this process will need to be considered.

## Conclusion

*Culex modestus* is well established in the ditch habitats that were surveyed at these sites across North Kent, and the results suggest that the species may well be found further east and west along the coastline as defined by suitable habitat. Initial surveys were made to identify suitable habitat further east, and whilst these surveys were not exhaustive, no suitable habitat was identified. This survey recorded a significant population of *Cx. modestus*, and given the likelihood of this species being found further afield, further surveys were conducted in 2014. These surveys have identified a wider distribution of the species, finding it as far west as Swanscombe (Gravesend, Kent), as far east as Canterbury (Kent), and also in East Tilbury and Pitsea (Essex) north of the Thames [21]. It is very likely that the species is found further afield, including further into Essex, and into Greater London as suitable habitats permit. Given a lack of historical survey data from many of these areas it is not possible to conclude whether the species has always been present, or has recently spread there. However, earlier, studies on the Hoo Penninsula near Cliffe Marshes and Northward Hill sites, and also at Elmley Marshes did not report the presence of this species [17,22]. The species has been reported to be highly impacted by anthropogenic environmental change in the Camargue, France [23], and in the Czech Republic it is now widely distributed and abundant having been found rarely in previous decades [24]. The species has also recently been reported for the first time in Denmark [25]. Within this context, the range and dominance of this species appears to be increasing in relation to other species in the UK. The study has further developed collection methods and assays for pathogen surveillance in mosquitoes in a UK context, and further work will aim to continue to develop this capability. Mosquito surveillance is an important addition to surveillance in wild birds, horses, and humans. This study also demonstrates a ‘One Health’ approach to zoonotic disease surveillance in the UK by integrating public health, veterinary health and academia.

As the principal bridge vector identified in European WNV cycles, the abundance of *Cx. modestus* together with populations of the enzootic vector *Cx. pipiens* s.l. in extensive habitats supporting resident and migratory birds is an important finding when considering the potential for WNV transmission in southern England. Abundant *Cx. modestus* populations in wetland areas with large avian populations, particularly migratory birds, and co-incident with livestock and horses are ecosystems at increased risk of WNV introduction and maintenance [26]. This survey suggests that WNV and USUV are not currently present in wetland sites in South-East England. However, the conditions for virus introduction are present in these areas and the spread of both viruses in Europe in recent years suggests that further monitoring is advisable.
